# Core protocol for the adaptive Platform Trial In COVID-19 Vaccine priming and BOOsting (PICOBOO)

**DOI:** 10.1186/s13063-023-07225-z

**Published:** 2023-03-18

**Authors:** C. McLeod, J Ramsay, K. L. Flanagan, M. Plebanski, H. Marshall, M. Dymock, J. Marsh, M. J. Estcourt, U. Wadia, P. C. M. Williams, M. C. Tjiam, C. Blyth, K. Subbarao, S. Nicholson, S. Faust, R. B. Thornton, A. Mckenzie, T. L Snelling, P. Richmond

**Affiliations:** 1 Wesfarmers Centre of Vaccines and Infectious Diseases, Telethon Kids Institute, Nedlands, Australia; 2 Centre for Child Health Research, The University of Western Australia, Crawley, Australia; 3 Infectious Diseases Department, Perth Children’s Hospital, Nedlands, Australia; 4 Tasmanian Vaccine Trial Centre, Clifford Craig Foundation, Launceston General Hospital, Launceston, TAS Australia; 5 School of Health Sciences, College of Health and Medicine, University of Tasmania, Launceston, TAS Australia; 6 School of Health and Biomedical Sciences, Royal Melbourne Institute of Technology University (RMIT), Melbourne, VIC Australia; 7 Women’s and Children’s Health Network, North Adelaide, Australia; 8 Robinson Research Institute and Adelaide Medical School, The University of Adelaide, Adelaide, Australia; 9 Sydney School of Public Health, Faculty of Medicine and Health, University of Sydney, Camperdown, Australia; 10 Department of Immunology and Infectious Diseases, Sydney Children’s Hospital Network, Westmead, Australia; 11 School of Women and Children’s Health, UNSW, Kensington, Australia; 12 Division of Paediatrics, School of Medicine, University of Western Australia, Crawley, Australia; 13 WHO Collaborating Centre for Reference and Research On Influenza, University of Melbourne, Parkville, VIC Australia; 14 Serology Laboratory, Victorian Infectious Diseases Research Laboratory, Melbourne, Australia; 15 Southampton Clinical Research Facility and Biomedical Research Centre, National Institute of Health Research, University Hospital Southampton NHS Foundation Trust, Southampton, UK; 16 Faculty of Medicine and Institute for Life Sciences, University of Southampton, Southampton, UK; 17 General Paediatrics and Immunology Departments, Perth Children’s Hospital, Nedlands, Australia

**Keywords:** COVID-19, Booster vaccination, Vaccination, Immunisation, Adaptive platform trial, Policy, Pandemic

## Abstract

**Background:**

The need for coronavirus 2019 (COVID-19) vaccination in different age groups and populations is a subject of great uncertainty and an ongoing global debate. Critical knowledge gaps regarding COVID-19 vaccination include the duration of protection offered by different priming and booster vaccination regimens in different populations, including homologous or heterologous schedules; how vaccination impacts key elements of the immune system; how this is modified by prior or subsequent exposure to severe acute respiratory syndrome coronavirus 2 (SARS-CoV-2) and future variants; and how immune responses correlate with protection against infection and disease, including antibodies and effector and T cell central memory.

**Methods:**

The Platform Trial In COVID-19 priming and BOOsting (PICOBOO) is a multi-site, multi-arm, Bayesian, adaptive, randomised controlled platform trial. PICOBOO will expeditiously generate and translate high-quality evidence of the immunogenicity, reactogenicity and cross-protection of different COVID-19 priming and booster vaccination strategies against SARS-CoV-2 and its variants/subvariants, specific to the Australian context. While the platform is designed to be vaccine agnostic, participants will be randomised to one of three vaccines at trial commencement, including Pfizer’s Comirnaty, Moderna’s Spikevax or Novavax’s Nuvaxovid COVID-19 vaccine. The protocol structure specifying PICOBOO is modular and hierarchical. Here, we describe the Core Protocol, which outlines the trial processes applicable to all study participants included in the platform trial.

**Discussion:**

PICOBOO is the first adaptive platform trial evaluating different COVID-19 priming and booster vaccination strategies in Australia, and one of the few established internationally, that is designed to generate high-quality evidence to inform immunisation practice and policy. The modular, hierarchical protocol structure is intended to standardise outcomes, endpoints, data collection and other study processes for nested substudies included in the trial platform and to minimise duplication. It is anticipated that this flexible trial structure will enable investigators to respond with agility to new research questions as they arise, such as the utility of new vaccines (such as bivalent, or SARS-CoV-2 variant-specific vaccines) as they become available for use.

**Trial registration:**

Australian and New Zealand Clinical Trials Registry ACTRN12622000238774. Registered on 10 February 2022.

## Administrative information

Note: The numbers in curly brackets in this protocol refer to the SPIRIT checklist item numbers. The order of the items has been modified to group similar items (see http://www.equator-network.org/reporting-guidelines/spirit-2013-statement-defining-standard-protocol-items-for-clinical-trials/).

**Table:** 

Title {1}	Core Protocol for the Adaptive Platform Trial In COVID-19 Vaccine priming and BOOsting (PICOBOO)
Trial registration {2a and 2b}.	Australian and New Zealand Clinical Trials Register ACTRN12622000238774; registered on 10/02/2022
Protocol version {3}	Core Protocol Version 11.0_16012023 Laboratory Appendix V4.0_16012023 Statistical Appendix V7.0_16012023
Funding {4}	Funding is provided by the Snow Foundation and the Medical Research Future Fund (MRFF): #2,014,690 and 2,016,473. These parties played no role in the design of the study nor the collection, analysis, or interpretation of the data or manuscript preparation.
Author details {5a}	McLeod C^1−3^, Ramsay J^1,3^, Flanagan KL^4−6^, Plebanski M^6^, Marshall H^7−8^, Dymock M^1,3^, Marsh J^1^, Estcourt MJ^9^, Wadia U^1−3^, Williams PCM^9−11^, Tjiam MC^1−2^, Blyth C^1−3,12^, Subbarao K^13^, Nicholson S^14^, Faust S^15^, Thornton RB^1−2^, Mckenzie A^1^, Snelling T^4^, Richmond P^1−2,12, 16^ ^1^Wesfarmers Centre of Vaccines and Infectious Diseases, Telethon Kids Institute, Nedlands, Australia ^2^Centre for Child Health Research, The University of Western Australia, Crawley, Australia ^3^Infectious Diseases Department, Perth Children’s Hospital, Nedlands, Australia ^4^Tasmanian Vaccine Trial Centre, Clifford Craig Foundation, Launceston General Hospital, Tasmania, Australia ^5^School of Health Sciences, College of Health and Medicine, University of Tasmania, Launceston, Tasmania, Australia ^6^School of Health and Biomedical Sciences, Royal Melbourne Institute of Technology University (RMIT), Melbourne, Victoria, Australia ^7^Women’s and Children’s Health Network, North Adelaide, Australia ^8^Robinson Research Institute and Adelaide Medical School, The University of Adelaide ^9^Sydney School of Public Health, Faculty of Medicine and Health, University of Sydney, Australia ^10^Department of Immunology and Infectious Diseases, Sydney Children’s Hospital Network, Australia ^11^School of Women and Children’s Health, UNSW, Australia ^12^Division of Paediatrics, School of Medicine, University of Western Australia, Crawley, Australia ^13^WHO Collaborating Centre for reference and research on Influenza, Department of Microbiology and Immunology, University of Melbourne, Melbourne, Victoria ^14^Serology Laboratory, Victorian Infectious Diseases Research Laboratory, Melbourne, Australia ^15^National Institute of Health Research Southampton Clinical Research Facility and Biomedical Research Centre, University Hospital Southampton NHS Foundation Trust, Southampton, United Kingdom ^16^Faculty of Medicine and Institute for Life Sciences, University of Southampton, United Kingdom ^17^General Paediatrics and Immunology Departments, Perth Children’s Hospital, Nedlands, Australia
Name and contact information for the trial sponsor {5b}	Trial Sponsor: Telethon Kids Institute (TKI), Northern Entrance, Perth Children’s Hospital, 15 Hospital Avenue, Nedlands, Australia Research Governance Office P: + 61 8 6319 1327
Role of sponsor {5c}	TKI is the sponsor and assumes ultimate responsibility for the initiation, conduct, management and financing of PICOBOO and the quality and integrity of study data. TKI delegates its sponsor study-related duties to the PICOBOO Trial Steering Committee, as stipulated in this protocol. TKI is providing clinical trial indemnity, and sites are providing site-specific indemnity for study personnel and equipment.

## Introduction

### Background and rationale {6a}

The coronavirus 2019 (COVID-19) pandemic represents an ongoing threat to global health [1]. While severe acute respiratory syndrome coronavirus 2 (SARS-CoV-2) is destined to become endemic, it is hard to anticipate how the transition to stable endemicity will unfold [2]. The durability of protection offered by vaccination is likely to be influenced by the host (including prior exposure to SARS-CoV-2), the evolution of the virus [1], the vaccine dose, the dosing interval and type of vaccines administered and the differential impacts of homologous versus heterologous schedules on immune responses (antibodies, B cells, CD4^+^ and CD8^+^ T cells) [3]. = Despite extensive research investment, critical uncertainties about the optimal strategies for COVID-19 priming and booster vaccination remain [4]. These include the duration of protection offered by different COVID-19 priming and booster vaccination strategies in different populations, especially children; how vaccination impacts key elements of systemic and mucosal immunity; how this is modified by prior or subsequent exposure to SARS-CoV-2 infection or homologous or heterologous vaccines at varying dosing intervals; and how these immune responses correlate with protection against infection and disease—especially against future variants/subvariants of concern (VoC) [4].

The Platform Trial In COVID-19 priming and BOOsting (PICOBOO) is a multi-site Bayesian, adaptive, randomised controlled, platform trial designed to simultaneously generate and translate high-quality evidence to fill critical knowledge gaps regarding the impacts of COVID-19 vaccination and natural infection on the immune system, to inform immunisation practice and policy. PICOBOO has been designed in consultation with the Australian COVID-19 Community Reference Group (CRG) and members of national COVID-19 policymaking bodies.

**Fig. 1:**
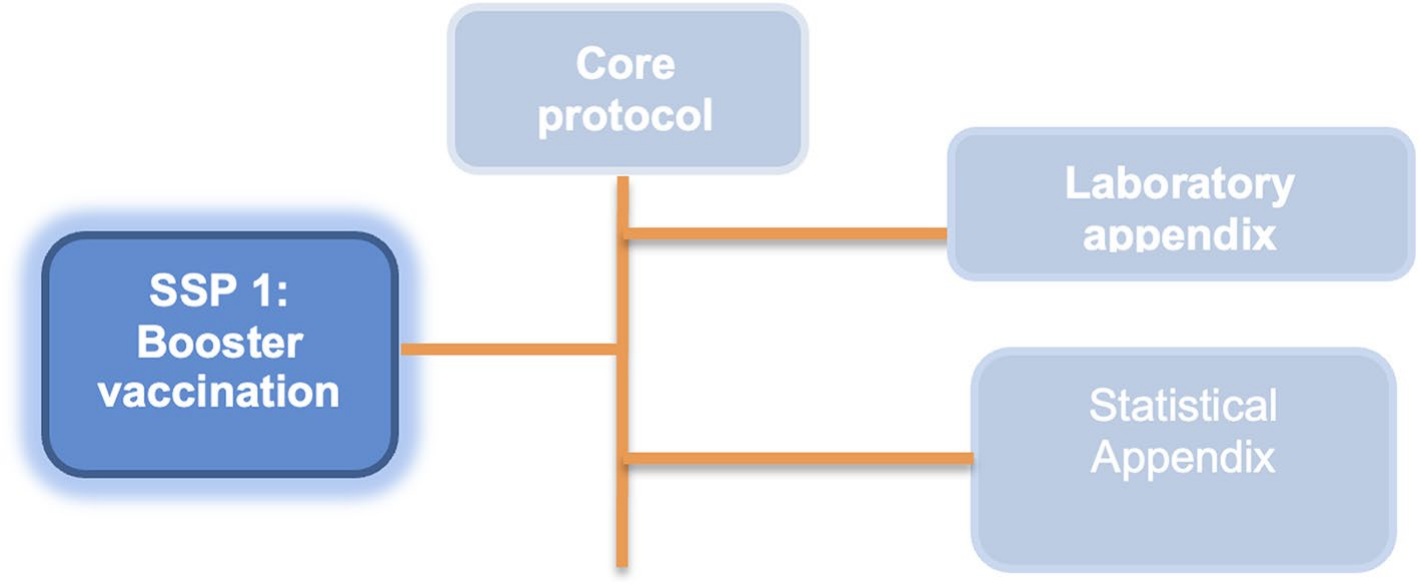
Modular PICOBOO protocol structure

The documentation specifying PICOBOO is modular and hierarchical (Fig. 1). Here, we present the Core Protocol, which describes the study procedures that apply to all participants and aspects of the trial, while a Substudy Specific Protocol (SSP) applies to describe study procedures that relate to participants enrolled in a specific nested substudy. This structure is intended to standardise outcomes, endpoints, data collection and other study processes for nested substudies included in the trial platform and to minimise duplication. SSPs and the statistical appendix are beyond the scope of this manuscript and will be detailed separately. The laboratory appendix is available from the corresponding author upon request.

### Objectives {7}

The primary objective is to generate high-quality evidence of the immunogenicity of different COVID-19 vaccination strategies against SARS-CoV-2 and its circulating variants/subvariants in immunocompetent children and adults, stratified by age group ± priming COVID-19 vaccination history.

The secondary objectives are to:

1. Compare the impacts of alternative COVID-19 booster vaccine strategies on elements of the immune system including the magnitude and breadth of SARS-CoV-2-specific B and T cell responses up to 720 days after randomisation
2. Compare the safety and reactogenicity of COVID-19 booster vaccine strategies against SARS-CoV-2 up to 28 days after randomisation
3. Compare the protection offered by different COVID-19 booster vaccine strategies, up to 720 days after randomisation, against (i) infection with SARS-CoV-2 or its variants/subvariants, (ii) hospitalisation due to disease and (iii) participant and carer reported time off work, study, or usual activities.
4. Harmonise and prospectively aggregate data generated by PICOBOO with international data to explore and define correlates of protection against COVID-19 infection and disease.

### Trial design {8}

A multi-site, multi-arm, Bayesian adaptive randomised, controlled, platform trial. PICOBOO will have the flexibility to introduce new vaccines/schedules for evaluation in this platform, or to remove arms in response to the emergence of external data or changes to immunisation policy in Australia, while preserving the integrity of the analyses.

## Methods: participants, interventions and outcomes

### Study setting [5]

This study will be conducted at three recruiting sites across Australia in the first instance; these include Telethon Kids Institute (Western Australia), the Women’s and Children’s Hospital (South Australia) and Launceston General Hospital (Tasmania).

### Eligibility criteria [5]

Eligibility criteria are defined at a platform and substudy level.

To be eligible for the platform trial, a person ± their guardian must:

1. Be ≥ 6 months of age
2. Be willing to receive a COVID-19 vaccine authorised for use (including emergency use authorisation or approved under a Clinical Trial Notification) by the Therapeutic Goods Administration (TGA) or equivalent regulatory authority
3. Be willing to comply with trial requirements, including the provision of biospecimens, at the indicated time intervals listed in the relevant SSP

A person is not eligible for the platform if they:

1. Are severely immunocompromised/have known immunodeficiency

### Who will take informed consent? {26a}

Informed consent will be obtained by written or electronic signature prior to enrolment by a member of the research team who has undergone Good Clinical Practice (GCP) training. A copy of the participant information and consent form (PICF; ≥ 12 years of age) will be provided to the participant detailing no less than the exact nature of the study, what is involved for the participant, the implications and constraints of the protocol and any risks and potential benefits associated with taking part and the storage and transfer of biological samples between participating research laboratories and retention of residual saliva and blood samples. Specific PICFs will be provided to children. Participation will be voluntary, and participants will be free to withdraw at any time, for any reason, without prejudice to future care and with no obligation to provide an explanation for withdrawal. For participants whose primary language is a language other than English (LOTE), informed consent will be obtained in conjunction with an accredited interpreter. An independent adult witness will be required to verify the consent process for LOTE participants.

### Additional consent provisions for collection and use of participant data and biological specimens {26b}

Consent for any data and/or biospecimens collected during this study to be used for future research purposes may also be invoked or withdrawn by the participant, their guardian or legal representative over time.

### Interventions

#### Explanation for the choice of comparators {6b}

All COVID-19 vaccines proposed for evaluation in the PICOBOO platform have, at a minimum, met the criteria for provisional registration (including emergency use) in Australia by the Therapeutic Goods Administration (TGA) or equivalent regulatory authority.

#### Intervention description {11a}

Eligible participants will be randomised to undergo COVID-19 vaccination according to the priming or booster vaccination schedule(s) stipulated in the relevant SSP. While the platform is designed to be brand-agnostic, the vaccines that will be evaluated at the commencement of the trial will include Pfizer’s Comirnaty® (Pf), Novavax’s Nuvaxovid® (Nx) and Moderna’s Spikevax® (Mod). If the vaccines studied at the commencement of the trial are superseded by alternatives, such as bivalent vaccines, a decision to substitute the vaccine intervention(s) with newer alternatives may occur, at the discretion of the trial steering committee (TSC). A maximum of three interventions per stratum will be evaluated at any given time.

#### Criteria for discontinuing or modifying allocated interventions {11b}

Withdrawal of participants from the study will occur if a participant or legal guardian requests withdrawal or if the treating clinician considers that ongoing participation is not in the best interest of the participant. If a participant withdraws from the study, storage of samples will continue, unless the participant requests otherwise. Any data collected before the withdrawal of the participant will still be used in the analysis in order to maintain safety and trial integrity. If a participant withdraws due to what they perceive to be an intolerable adverse event (AE), they will be given appropriate immediate treatment and returned to the care of their treating physician.

Recruitment to a particular stratum will be ceased if the pre-specified decision criteria threshold is met (detailed in the “Statistical methods for primary and secondary outcomes {20a}” section).

#### Strategies to improve adherence to interventions {11c}

Different vaccination strategies are being evaluated in this trial. Therefore, strategies to improve adherence are not relevant.

#### Relevant concomitant care permitted or prohibited during the trial {11d}

Routine or emergency medical care will not be impacted by participation in the PICOBOO trial.

#### Provisions for post-trial care {30}

Participants in the PICOBOO trial will receive usual supportive care following vaccination, as per standard Australian immunisation practice. Specifically, participants will be observed for a minimum of 15 min, and supportive treatment for the management of acute hypersensitivity reactions (e.g. anaphylaxis) will be administered, if required.

### Outcomes {12}

The primary estimand (inferential target for estimation) for all participants, is defined by:

1. *Study population*: Participants ≥ 6 months of age willing to undergo COVID-19 vaccination with vaccines authorised for use (including emergency use or approved under a clinical trial notification).
2. *Intervention*: Prescription of a COVID-19 vaccination intervention within a stratum, defined in the relevant SSP.
3. *Endpoint*: The amount of binding antibody, measured as the geometric mean concentration of ancestral SARS-CoV-2 anti-spike immunoglobulin G (IgG) ~ 28 days after randomisation.
4. *Primary effect measure*: The geometric mean concentration of ancestral SARS-CoV-2 anti-spike IgG for each intervention in each stratum and separately for each SARS-CoV-2 vaccination intervention, adjusting for any differences in baseline factors between intervention arms that might otherwise confound or reduce the certainty of the treatment effect.
5. *Intercurrent events*: This includes events that occur after randomisation that might confound or otherwise impact the measure of the treatment effect of the intervention. Common anticipated intercurrent events include (i) non-adherence to the allocated intervention due to site procedures or availability; (ii) non-adherence to the allocated intervention due to a serious adverse event (SAE) or perceived clinical indication; (iii) missing blood sample within the appropriate window for endpoint collection, excluding those missed due to adverse events (AE)/SAE but including withdrawals and participants lost to follow-up; (iv) missing blood sample within the appropriate window due to AE/SAE but excluding withdrawals and lost to follow-up; (v) evidence of SARS-CoV-2 infection: rapid antigen test (RAT), polymerase chain reaction (PCR) or anti-nucleocapsid antibody seroconversion between randomisation and day ~ 28; and (vi) evidence of a further SARS-CoV-2 vaccine dose following randomisation. Intercurrent events strategies are beyond the scope of this document and will be published separately in a statistical appendix.

#### Immunogenicity

The timing of immunological endpoints will vary depending on age strata and will be specified in the relevant SSP. A summary of endpoints reported for all participants included in the platform trial is presented in Table 1. Endpoints will be reported for each intervention in each stratum. Additional endpoints may be reported for individual nested substudies; these will be detailed in the relevant SSP.

**Table 1:**
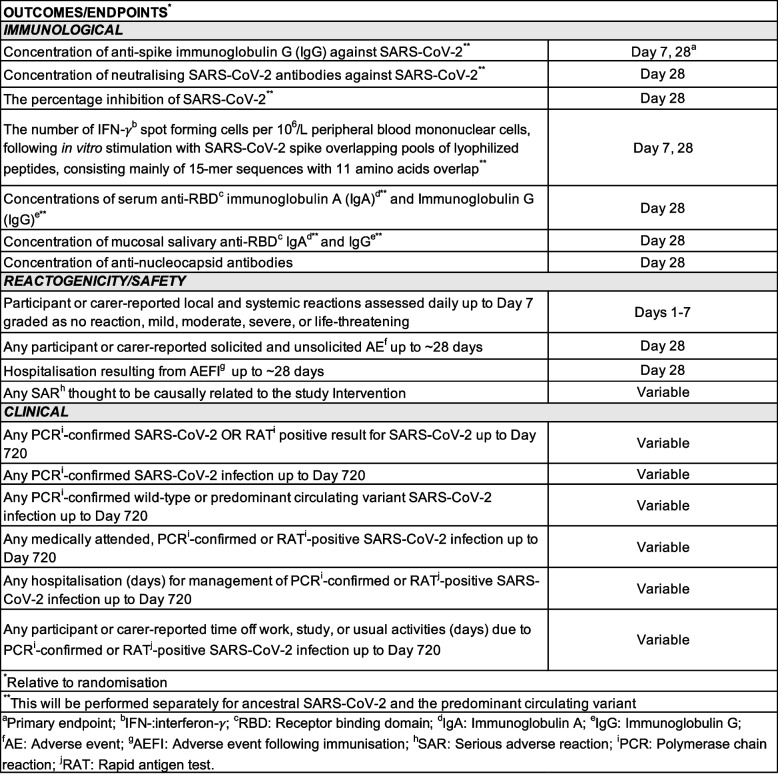
Outcomes and endpoints for the PICOBOO platform trial

#### Participant timeline {13}

Study visits for participants within individual strata will be depicted in the relevant SSP.

#### Sample size {14}

This platform trial is designed to be a perpetual trial recruitment platform; therefore, no fixed sample size is specified.

#### Recruitment {15}

Potential screening avenues to identify eligible participants include:

- *During immunisation clinic visits*—potential participants may be directly approached during scheduled immunisation visits at participating sites. Trained research staff will approach participants to discuss participation.
- *Advertising material*—including flyers or bulletins may be used to promote and raise awareness; these may appear on public notice boards or electronic notice boards. Advertisements may also be displayed in electronic or print media and various community locations.
- *Text/email message*—potential participants may be provided with a link to the study advertising material, within their vaccine appointment reminder, and be invited to register their interest within the study REDCap database.
- The trial sponsor and external recruitment agencies will assist with achieving targeted recruitment to the pre-specified strata. This will be achieved via multiple advertising channels using multimedia, including texts/email messages/letters and public or electronic flyers, newspapers or bulletins on notice boards and social media (including Facebook and Twitter).

The research staff at the participating sites will receive training regarding study requirements to ensure eligible participants are identified, provided with relevant information, and invited to participate in the study. Site agreements will stipulate that enrolment and screening procedures occur according to the approved protocol and are in line with the principles of GCP and the Declaration of Helsinki.

Each site will maintain a screening log to capture basic demographic data on all individuals screened, eligible and enrolled and reasons for non-eligibility. The purpose of the log is to allow full characterisation and representativeness of the study population and to identify any deficiencies in the process.

Recruitment strategies will use approved material and may be broad to target the overall study population or specific subgroups of participants based on age and COVID-19 vaccination history.

### Assignment of interventions: allocation

#### Sequence generation {16a}

A sequence of intervention assignments will be generated by an unblinded trial statistician using random permuted blocks for each stratum using computer software with a validated random number generator and equal allocation for all vaccination interventions. These allocations will be specified in the relevant SSP.

#### Concealment mechanism {16b}

On the day of COVID-19 vaccination, participants for whom all eligibility criteria have been satisfied, informed consent for participation has been obtained and a unique participant number has been generated from the REDCap Database, will be randomised. A qualified and delegated member of the research team will record the participant’s identifiers and establish the appropriate stratum. Randomisation will then be performed within the REDCap database where the allocation sequence is inaccessible and adequately concealed from the research team.

#### Implementation {16c}

At vaccination, two unblinded members of the research team will check and dispense the study vaccine for administration. Pre-filled syringes containing one of the COVID-19 booster vaccines approved for use will be covered with opaque tape and concealed until ready for administration. Prior to opening the box, the participant will be asked to look away. The vaccine intervention will be administered in accordance with routine immunisation practices stipulated per Australian guidelines.

### Assignment of interventions: blinding

#### Who will be blinded {17a}

Participants will be blinded to the specific COVID-19 vaccination received, at least until after the primary estimand data are collected (see the “Procedure for unblinding if needed {17b}” section). The research staff involved in the follow-up of adverse reactions will be blinded to the group assignment of the participants. The laboratory staff processing or analysing trial specimens will be blinded to the group assignment of participants. Researchers involved with the trial design adaptations will be blinded to trial results until the first interim analysis.

Trial analysts preparing interim reports will be unblinded. The DSMC will receive open (unblinded) safety data reports at regular interims.

#### Procedure for unblinding if needed {17b}

The corresponding site principal investigator will provide authorisation for unblinding if compelling reasons arise, such as if this information is required for visa or employment purposes. Authorisation for unblinding will be communicated to the unblinded trial statistician. Participants for whom the assignment is revealed will remain in the study and where possible continue to receive surveys and attend scheduled study visits.

All vaccinations administered to participants over the course of the study will be recorded on the Australian Immunisation Record (AIR) by the unblinded vaccine nurse approximately 6 weeks following randomisation (to ensure unbiased reporting of the primary estimand and safety outcomes).

### Data collection and management

#### Plans for assessment and collection of outcomes {18a}

Data will be collected on hard or electronic case report forms (eCRF), including (i) demographic data, (ii) COVID-19 vaccination history, (iii) previous medical history, (iv) anthropometric data (including height and weight), (v) laboratory data and (vi) patient ± carer-reported outcomes (see Table 2).

**Table 2:**
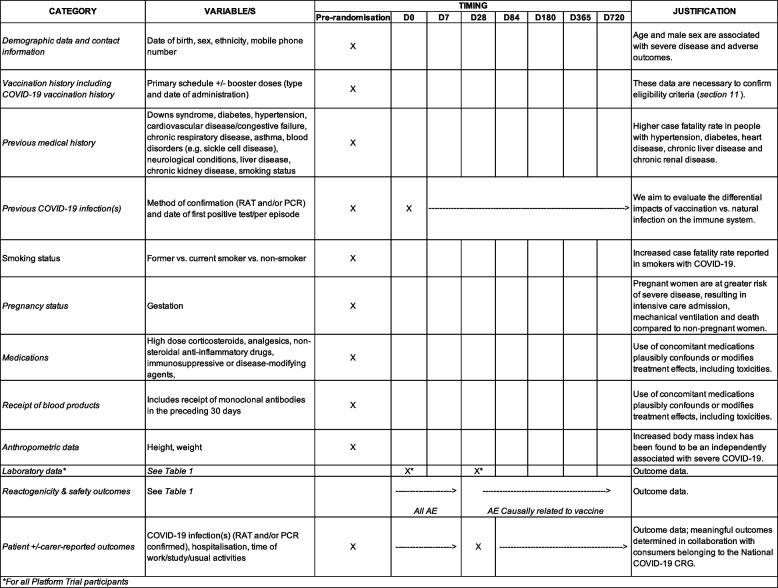
Outcome data for PICOBOO participants

#### Plans to promote participant retention and complete follow-up {18b}

Participants will be provided with electronic prompts and reminders to complete self-reported outcome data. Follow-up phone calls and reminder cards will be provided to minimise missing data and missed appointments. Participants will also be financially remunerated for their contribution to the study if they choose to participate.

Data quality and adherence to the protocol will be promoted by (i) conducting a commencement meeting for all project coordinators and investigators at each site prior to their involvement, (ii) performing site inductions which includes specific training about the protocol and the provision of reference documents for project staff, (iii) provision of a data dictionary to assist with data entry on the eCRF and (iv) timely validation of data by the data management team.

#### Data management {19}

Data will be collected into a secure REDCap database, hosted by the sponsor. Data will be entered into an eCRF developed in accordance with the Core Protocol and relevant SSP. Data entry will be performed by trained study staff, apart from electronic patient-reported outcome data which will be directly entered by the participant.

Information will be recorded in the eCRF to accurately reflect the source data. All users will have appropriate permissions defined by their user role type which is delegated by the sponsor and site investigator.

Data entry and data management will be coordinated by the central data manager. The data manager and study coordinator, or delegate, will ensure the integrity of the data by performing regular and timely quality control checks. Corrections will be made if errors are identified during this process. Any common patterns of errors will be reported back to the participating sites. Missing data will be minimised through a clear and comprehensive data dictionary with online data entry including logic checks.

#### Confidentiality {27}

Participation in this study involves the potential risks of accidental breach of confidentiality of the recorded information and breach of the privacy of the participants. This will be minimised by removing direct participant identifiers (i.e. names, Medicare numbers, medical record numbers) from the information stored for the study and used for analysis, securing, and limiting access to linking codes assigned to the study information and limiting access to the information contained within the study to authorised research staff.

#### Plans for collection, laboratory evaluation and storage of biological specimens for genetic or molecular analysis in this trial/future use {33}

The collection, laboratory evaluation and storage of biological specimens for analysis from participants in the PICOBOO trial are outlined in the PICOBOO Laboratory Appendix, relevant SSP and the trial standard operating procedures (SOPs). Blood and saliva samples will be collected at pre-specified time points. Blood will be separated into the sera, plasma, and peripheral blood mononuclear cells (PBMCs) at local sites prior to transportation to reference laboratories for processing. Granulocytes collected from baseline blood samples during gradient centrifugation will be used for deoxyribonucleic acid (DNA) extraction for human leucocytic antigen (HLA) I and II typing. This will be performed to evaluate susceptibility to and protection from SARS-CoV-2 infection and disease. Genomic DNA will be extracted using commercial kits, harmonised across sites. DNA will be aliquoted and stored as per SOPs.

## Statistical methods

### Statistical methods for primary and secondary outcomes {20a}

A Bayesian three-level hierarchical linear model will be used for the primary analysis as it is anticipated that immune responses may be mutually informative across COVID-19 vaccination dose, age groups and potentially across messenger ribonucleic acid (mRNA) vaccine interventions (Pfizer Comirnaty and Moderna Spikevax). For the first level, information is borrowed across vaccination dose number within age groups, intervention allocation and vaccine history. For the second level, information is borrowed across age groups within intervention allocation and vaccine history. Finally, for the third level, information is borrowed across mRNA vaccine intervention allocation, for the ancestral subtypes only, within vaccine history. The model estimates the posterior distribution of the mean log_10_ anti-spike SARS-CoV-2 IgG antibody against Ancestral SARS-CoV-2 measured ~ 28 days after receipt of the assigned booster COVID-19 vaccine for each intervention and vaccination strategy in each stratum, denoted by vaccine history group and age group.

A detailed description of the statistical methods for this trial is beyond the scope of this document and will be reported separately as a statistical appendix plan. The SAP is available from the corresponding author upon request and will be available on the trial website, once live.

### Interim analyses {21b}

The first analysis will be performed after participants have completed 300 vaccination events and have completed 21–31 days of follow-up post-randomisation, and the results from the batched blood samples are available from the laboratory analysis; thereafter, analyses will be performed after every 150 additional vaccination occasions with available laboratory results for the remainder of the trial, unless otherwise stipulated in the statistical appendix.

### Methods for additional analyses (e.g. subgroup analyses) {20b}

The pre-specified mutually exclusive stratum for the primary and secondary analyses is specified in the relevant SSP.

Any endpoints not defined in the “Outcomes {12}” section will be designated as exploratory.

### Methods in analysis to handle protocol non-adherence and any statistical methods to handle missing data {20c}

Immune responses and reactogenicity to COVID-19 vaccines will be assessed using a treatment policy strategy. Further detail regarding the analytical approach to handling intercurrent events is detailed in the statistical appendix.

### Plans to give access to the full protocol, participant-level data and statistical code {31c}

The current version of the full PICOBOO protocol and the Statistical and Laboratory Appendices will be accessible on the trial website. Decisions regarding the sharing of de-identified data and/or statistical code will be assessed by the PICOBOO TSC and will be conditional upon any necessary institutional and ethics approvals.

### Oversight and monitoring

#### Composition of the coordinating centre and trial steering committee {5d}

**Fig. 2:**
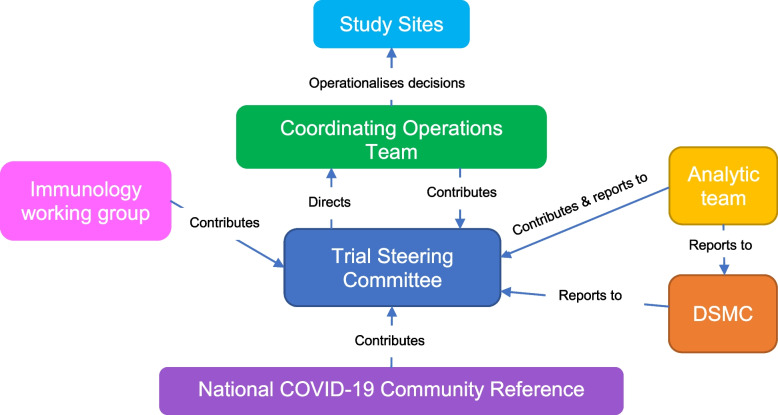
PICOBOO administrative structure

Please refer to Fig. 2 for an overview of the PICOBOO administrative structure. The roles and responsibilities for individuals and groups that sit within this administrative framework are outlined in their terms of reference.

#### Composition of the data monitoring committee, its role and reporting structure {21a}

A Data and Safety Monitoring Committee (DSMC) will be appointed to provide safety oversight. The DSMC will have an advisory role as outlined in the DSMC Charter. The DSMC will be notified of all SAEs in accordance with the DSMC charter and will undertake a timely review of these as per the Charter. The DSMC will inform the coordinating principal investigator (CPI) immediately to recommend termination of the study if deemed necessary following a serious adverse reaction (SAR). The DSMC will meet at least three times per year to monitor and review accumulating safety reports and will make recommendations to the TSC on whether there are any ethical or safety reasons why the trial should not continue or should be modified prior to continuation. The DSMC will consider:

- The occurrence and nature of adverse events
- Whether additional information on adverse events is required
- Taking appropriate action where necessary to halt the trial
- Incidents occurring between meetings that require rapid assessment (e.g. sudden unexpected serious adverse events (SUSARs))

Sequential analyses will be performed on the accumulating trial data for the primary estimand at pre-specified times detailed in the statistical appendix. The results of these analyses will be provided confidentially to the DSMC as an unblinded report, including recommendations to continue or stop recruitment into each stratum depending on whether the precision threshold has been met. All recommendations based on pre-specified criteria in the statistical appendix will be reviewed by the DSMC as specified in the DSMC charter. When a precision threshold is confirmed as having been reached by the DSMC and where no compelling reason exists not to reach a conclusion for a specific stratum or trial overall, a recommendation may be issued for a public disclosure of the result. Following any interim analysis, the DSMC will make recommendations to the CPI on whether there are any ethical or safety reasons why the trial should not continue.

The DSMC may request to be unblinded to the vaccine allocation of individual participants and/or to have blinded or unblinded analyses performed by the unblinded trial statistician.

The DSMC will comprise a minimum of three independent advisors, including at least one clinician expert in vaccinology, a person experienced in clinical trials and a statistician. The chair of the DSMC will be contacted for advice and independent review in the following situations:

- Following any SUSAR
- When a decision threshold is met
- If the investigator committee feels independent advice or review is important

#### Adverse event reporting and harms {22}

Safety reporting for the trial will commence once the first participant is enrolled and will end once the final study participant has completed their final survey on ~ day 720 (+ 56 days) after randomisation. All SAEs, AESI, medically attended AEs (MAAEs) and AEs resulting in withdrawal occurring from day 0 to day 28 after randomisation will be recorded in the eCRF. From day 29 to day 720 after randomisation, all SAEs, AESIs, MAAEs, and AEs resulting in withdrawal that are found to be related to the study vaccine or study procedures will be reported. After day 28, COVID-19 and associated events will be considered a clinical outcome, not an AESI. In addition, any solicited or unsolicited AE regardless of whether attributed to trial vaccination or not occurring daily during the 7 days after vaccination will also be recorded on the CRF. All solicited AEs that persist beyond day 7 or AEs that result in a participants’ withdrawal from the study at any time will be followed up until a satisfactory resolution occurs, or until a non-study related causality is assigned (if the participant consents to this).

All SAEs must be reported to the sponsor within 24 h of study staff awareness of the SAE. An initial SAE report form will be completed with as much information as is available at the time and signed by the principal investigator or delegate unless otherwise specified in the sponsor SOP. All SUSARs will be reported by the sponsor to the TGA. For fatal and life-threatening SUSARs, this will be done immediately but no later than 7 calendar days after the site study team is first aware of the reaction. Any additional relevant information will be reported within 8 calendar days of the initial report. All other SUSARs will be reported within 15 calendar days of being made aware of the event.

The sponsor is responsible for the ongoing safety evaluation of the investigational product. The sponsor maintains overall responsibility for generating safety communications generated through feedback from the DSMC.

#### Frequency and plans for auditing trial conduct {23}

Monitoring will be performed in accordance with GCP standards. Monitors will verify that PICOBOO data are generated, documented, and reported in compliance with the protocol, source documents and applicable regulatory requirements.

A risk-based approach has been used to develop a monitoring plan. This means that specific risks for this project have been identified and will inform routine monitoring of site-specific processes and complete source data verification. This has identified and defined the data and processes critical to data quality and the processes required to minimise them. Quality indicators and thresholds that would trigger an investigation and/or corrective action have been set.

Obligations expected of sites to assist the sponsor in monitoring the study may include hosting site visits, providing information for remote monitoring or putting procedures in place to monitor internally. If additional studies are nested or added to the PICOBOO platform trial, additional processes for defining, assessing, reporting, and monitoring these additional research activities will be outlined in their relevant SSPs.

#### Plans for communicating important protocol amendments to relevant parties (e.g. trial participants, ethical committees) {25}

Any substantial amendments to the PICOBOO protocol will require prior approval by the relevant ethics and governance regulatory bodies. A substantial amendment is defined as any change which impacts the conduct, management or scientific integrity of the study, the safety of participants or which involves a change to the interventions studied (including the addition or cessation or intervention arms) or the randomisation of interventions within a stratum. Minor amendments that do not meet these criteria will be noted and filed by the sponsor and will not require notification to the regulatory bodies. The trials registry will be updated on an ad hoc basis, if required.

#### Dissemination plans {31a}

The results will be communicated via the trial website and by presentations and peer-reviewed publications. The Steering Committee will coordinate the dissemination of information to relevant stakeholders, including immunisation policymakers. The National COVID-19 Consumer Reference Group will provide guidance on the best methods for the dissemination of information to participants and the broader community. The TSC will, as far as possible, make the protocol(s), statistical analysis plans, and non-identifying patient-level data available, to allow independent scientific scrutiny and validation of any published results. All investigators will have the opportunity to review publications (e.g. manuscripts, abstracts, oral/slide presentations, book chapters) prior to submission. Authorship will be determined in line with the uniform requirements for manuscripts submitted to biomedical journals published by the International Committee of Medical Journal Editors.

## Discussion

This document sets out a Core Protocol for PICOBOO; an adaptive platform trial designed to evaluate the immunogenicity, reactogenicity and cross-protection offered by different COVID-19 priming and booster vaccination strategies against SARS-CoV-2 and its variants/subvariants. Currently, it is unclear whether periodic boosting will be necessary in the future, and if so, whether this should be recommended for all Australians, or targeted to specific vulnerable populations or age groups. This trial is designed to generate high-quality evidence to inform Australian immunisation practice and policy, but with global relevance; this will be critical to achieving national and global control of COVID-19.

The primary outcome measure will be the geometric mean concentration of anti-spike IgG ~ 28 days after randomisation to the vaccination intervention. This endpoint has been chosen to align with Com-COV [6], COV-BOOST [7] and international COVID-19 vaccination trials, as well as Bringing Optimised COVID-19 Vaccine Schedules To ImmunoCompromised Populations (BOOST-IC; NCT05556720), which is a platform trial evaluating different booster vaccination strategies in immunocompromised hosts in Australia. Currently, there are no recognised correlates of protection against COVID-19 infection and disease, although recent studies indicate that anti-spike neutralising antibody levels likely correlate with vaccine-induced protection [8]. It is hoped that prospectively aggregated data generated by PICOBOO will add to the international databank to enable further exploration of the clinical correlates of COVID-19 infection and disease.

We have defined the statistical analyses for this trial using the estimands framework; this approach is recommended in the latest version of the ICH statistical guidelines [9]. Each estimand, defined as an inferential target for estimation, is defined by five key attributes. These include (i) the population of interest, (ii) the endpoint (analysed parameter), (iii) a description of the treatment intervention(s), (iv) intercurrent events that occur following randomisation that might impact on the treatment effect and (v) the population level summary [10]. This framework is designed to ensure that the objectives of the trial align with the design and conduct of the study and to improve transparency regarding the analysis and reporting of results, to improve the value of the research that is conducted.

This Core Protocol sets out the establishment of the first adaptive platform trial within Australia, and one of the few established platforms internationally, to enable the evaluation of different COVID-19 vaccination strategies, concurrently. It is envisaged that this platform will enable the evaluation of multiple COVID-19 vaccination interventions (e.g. different booster numbers) in various population cohorts and/or subgroups. The use of an adaptive platform design has the benefits of flexibility and efficiency compared to conventional trials. This platform will enable researchers to respond with agility to new policy questions as they arise, including the potential rollout of new vaccine formulations (such as bivalent formulations or vaccines targeted to specific variants) become new VoC arise in Australia, prompting evidence-based changes in strategy.

The overarching PICOBOO platform will facilitate separately funded, nested mechanistic substudies by established research networks, which will benefit from the established governance, research, and analytic personnel, recruiting sites and digital and trial infrastructure. This will allow sharing of resources and cost-savings across multiple distinct but closely related trials, which traditionally would have been conducted sequentially or in parallel, rather than simultaneously. It is envisaged that data collection, endpoints and methods for analysis and reporting of results will be standardised across these nested or closely related projects, where possible.

There are two major advantages in using this trial design and protocol structure. Firstly, borrowing information across statistical models when the treatment effect is similar in different strata will contribute information to inform an estimation of the posterior probability in another stratum. Secondly, this flexible trial structure will enable investigators to respond to new policy questions and vaccination interventions as they arise (for example, the impact of primary vaccination on younger age cohorts).

PICOBOO will establish a national biobank of serum, plasma, PBMCs and saliva for future use by Australian researchers. It will be possible to apply emerging technologies assessing cellular, humoral, and mucosal immunity against existing and future SARS-CoV-2 variants/subvariants using these banked samples.

Designed in consultation with the National COVID-19 Community Reference Group (CRG), PICOBOO will improve our understanding of the impacts of COVID-19 vaccination on humoral and cellular immunity, and thereby improve public confidence in the evidence-underscoring policy decisions, which will be critical to achieving national and global control of COVID-19.

## Trial status

Current protocol version 11.0 (16-01-2023). Recruitment commencement date: 29–03-2022. Recruitment is expected to be complete by 07–02-2025. The data that support the findings of this study may be available from the corresponding author, CM, upon reasonable request.

## Data Availability

Access to data will be granted to study investigators and authorised representatives from the sponsor and the regulatory authorities to allow trial-related monitoring, audits, and inspections to occur. PICOBOO will also comply with relevant jurisdictional and academic requirements relating to access to data, as applied at the time that the data are generated.

## Abbreviations

AE: Adverse event
AIR: Australian Immunisation Register
AZ: AstraZeneca
eCRF/CRF: (Electronic) case report form
COM-COV: Comparing COVID-19 vaccine schedule combinations
COVID-19: Coronavirus 2019
CPI: Coordinating principal investigator
COV-BOOST: Comparing COVID-19 booster vaccinations
CRG: Consumer Reference Group
DNA: Deoxyribonucleic acid
DSMC: Data Safety Monitoring Committee
HLA: Human leucocyte antigens
ICH GCP: International Committee for Harmonisation Good Clinical Practice
Ig: Immunoglobulin
Nx: Nuvaxovid Novavax
MAAE: Medically attended adverse events
MedRA: Medical Dictionary for Regulatory Authorities
Mod: Moderna Spikevax
PBMC: Peripheral blood mononuclear cells
PCR: Polymerase chain reaction
mRNA: Messenger ribonucleic acid
Pf: Pfizer Comirnaty
PICOBOO: Platform trial In COVID-19 BOOsting
RAT: Rapid antigen test
RBD: Receptor-binding domain
SARS-CoV-2: Severe acute respiratory syndrome coronavirus 2
SAE: Serious adverse event
SAR: Serious adverse reaction
SD: Standard deviation
SOP: Standard operating procedure
SUSAR: Sudden unexpected serious adverse reaction
TKI: Telethon Kids Institute
TGA: Therapeutic Goods Administration
TMP: Trial Master Protocol
TSC: Trial Steering Committee
VoC: Variants of concern
